# Production of Recombinant Peanut Allergen Ara h 2 using *Lactococcus lactis*

**DOI:** 10.1186/1475-2859-6-28

**Published:** 2007-08-21

**Authors:** Jacob Glenting, Lars K Poulsen, Kentaro Kato, Søren M Madsen, Hanne Frøkiær, Camilla Wendt, Helle W Sørensen

**Affiliations:** 1 Bioneer A/S, DK-2970 Hørsholm, Denmark; 2 Allergy Clinic 7751, National University Hospital, DK-2100 Copenhagen, Denmark; 3 Department of Medical Biochemistry and Genetics, University of Copenhagen, DK-2200 Copenhagen, Denmark; 4 Biocentrum DTU, DK-2800 Kgs. Lyngby, Denmark

## Abstract

**Background:**

Natural allergen sources can supply large quantities of authentic allergen mixtures for use as immunotherapeutics. However, such extracts are complex, difficult to define, vary from batch to batch, which may lead to unpredictable efficacy and/or unacceptable levels of side effects. The use of recombinant expression systems for allergen production can alleviate some of these issues. Several allergens have been tested in high-level expression systems and in most cases show immunereactivity comparable to their natural counterparts. The gram positive lactic acid bacterium *Lactococcus lactis *is an attractive microorganism for use in the production of protein therapeutics. *L. lactis *is considered food grade, free of endotoxins, and is able to secrete the heterologous product together with few other native proteins. Hypersensitivity to peanut represents a serious allergic problem. Some of the major allergens in peanut have been described. However, for therapeutic usage more information about the individual allergenic components is needed. In this paper we report recombinant production of the Ara h 2 peanut allergen using *L. lactis*.

**Results:**

A synthetic ara h 2 gene was cloned into an *L. lactis *expression plasmid containing the P170 promoter and the SP310mut2 signal sequence. Flask cultures grown overnight showed secretion of the 17 kDa Ara h 2 protein. A batch fermentation resulted in 40 mg/L recombinant Ara h 2. Purification of Ara h 2 from the culture supernatant was done by hydrophobic exclusion and size separation. Mass spectrometry and N-terminal analysis showed a recombinant Ara h 2 of full length and correctly processed by the signal peptidase. The immunological activity of recombinant Ara h 2 was analysed by ELISA using antibodies specific for native Ara h 2. The recombinant Ara h 2 showed comparable immunereactivity to that of native Ara h 2.

**Conclusion:**

Recombinant production of Ara h 2 using *L. lactis *can offer high yields of secreted, full length and immunologically active allergen. The *L. lactis *expression system can support recombinant allergen material for immunotherapy and component resolved allergen diagnostics.

## Background

The objective of allergen immunotherapy is to counteract an already established pathological immune response against the administered protein. The most frequently used form in the clinic is specific immunotherapy, which involves repeated subcutaneous injection of increasing doses of adjuvant-bound allergen extract [1]. Recently, needle free and mucosal vaccination such as sublingual administration has been successfully exploited using allergens from house dust mite and cat dander [2] and the grass allergen Phl p 5 [3,4]. Allergen immunotherapy relies on repeated immunizations for a relative long period. The therapeutic strategy, particularly the sublingual variant, requires therefore relatively large amounts of allergen and demands high quality standards of the source of allergen. Most therapies use allergen extracts from natural sources, which contain the native (iso)forms of the proteins. Crude extracts prepared from natural sources can however be difficult to standardise and contain difficult to define mixtures of allergens (reviewed by [5]). In addition to the protein allergens, they also contain non-allergenic proteins and other substances.

Recombinant produced allergens may increase the safety of immunotherapy and overcome some of the problems associated with natural allergen extracts [6]. The most important allergens have been cloned and sequenced. The use of these genes for recombinant allergen expression can facilitate *i*) high yield allergen production with low biological or batch to batch variation *ii*) material for refined and component-resolved allergy diagnosis *iii*) allergen preparations of defined purity and composition *iv*) development of engineered hypoallergens that show reduced binding to IgE. The drawbacks of recombinant production are associated with lack of product-authenticity and that some therapies require multiple allergens, some of which are yet unknown. High-level expression systems for production of allergens have been developed. These are based on either bacteria or eukaryotes. The birch pollen Bet v 1 allergen has been produced using the T7 based *Escherichia coli *system with a yield of 8–10 mg purified allergen per litre culture [7]. Plants have also been tested as recombinant allergen factories. The olive pollen allergen, Ole e 3 and Ole e 8, was produced in *Arabidopsis thaliana *and showed similar biological activities as their natural counterpart [8]. The choice of recombinant expression system for allergen production is a balance between product yield, authenticity and immunereactivity, and cost effectiveness. In most cases, the immunereactivity of recombinant allergens is comparable with their natural counterparts (reviewed by [9]). Microbial based expression systems are simple and cost effective. However, more complicated and eukaryotic based expression systems are necessary where post translational modifications like glycosylation play an essential role in the allergenicity of the protein. An example is the Cit s 1 from oranges bearing a single N-glycan, which is the target of the IgE response to this protein [10]. Recombinant systems with differing post translational machineries may therefore produce allergens with same amino acid composition, but with different allergenecity. Therefore, different expression systems have been compared. The storage mite allergen Lep d 2, causing disease amongst farmers, was produced in *E. coli *and adherent cell cultures at yields of 1 mg/L and 4 mg/L, respectively [11]. Both types of recombinant Lep d 2 products showed immune-reactivity similar to that of natural Lep d 2 when tested against patient sera. Interestingly, this indicates that IgE epitopes are retained in the recombinant proteins, each produced by two very different expression hosts.

Recombinant production of genetically engineered hypoallergens offers a potential improvement of immunotherapy. By site directed mutagenesis, allergens with epitopes that display reduced IgE binding, but retained T-cell epitopes, can be developed [12]. The approach was first developed for dust mite allergen Der p 2 where engineered protein variants without disulfide bonds showed reduced IgE binding [13]. A recombinant engineered hypoallergen variant of Bet v 1 was better tolerated compared to its natural counterpart in a clinical trial with allergic patients [14]. It is generally accepted that engineered hypoallergens opens for immunotherapy with higher, but still safe, doses of allergen.

Peanut allergy is one of the most severe food allergies with a prevalence of 0.6% in UK, USA and Australia [15-17]. Treatment relies today on strict avoidance of peanuts in the diet and ready-to-access self injectable epinephrine. The major allergens in peanut have been identified as Ara h 1–3 [18], where Ara h 2 is the most frequently recognised [19]. More than 90% of the patients allergic to peanut have IgE specific to Ara h 2 [20]. The gene encoding Ara h 2 has been used for plasmid DNA vaccination. Oral administration of chitosan formulated plasmid DNA induced Ara h 2 expression in the intestinal epithelium and reduced Ara h 2 specific IgE activity in a mouse model [21]. Although direct inoculation of plasmid DNA into the patient is a simple vaccine strategy, the technique still suffers from efficacy and safety issues [22]. Recombinant engineering and production of the Ara h 2 protein has also been tested. A hypoallergen variant of Ara h 2 with altered IgE epitopes showed reduced IgE-binding compared to the wild type Ara h 2 [23]. Expression of Ara h 2 in *E. coli *showed similar conformational features of the heterologous and native Ara h 2 product [24]. The *E. coli *produced Ara h 2 was however accumulated intracellularly and an affinity-tag was added to the N-terminal of the protein to facilitate purification.

The present study tested the use of the lactic acid bacteria *Lactococcus lactis *as microbial production host of the major peanut allergen Ara h 2. *L. lactis *is a gram positive bacterium with food grade status due to its long history of use in the manufacturing of dairy products. We used a plasmid based and secretory expression system [25] for production of recombinant Ara h 2 (rAra h 2) by 1 litre batch fermentation and a simple purification protocol. The rAra h 2 was characterized by mass spectrometry, N-terminal sequencing and immunological analysis.

## Results

### Expression and purification of rAra h 2 allergen using *L. lactis *in batch fermentation

**Figure 1:**
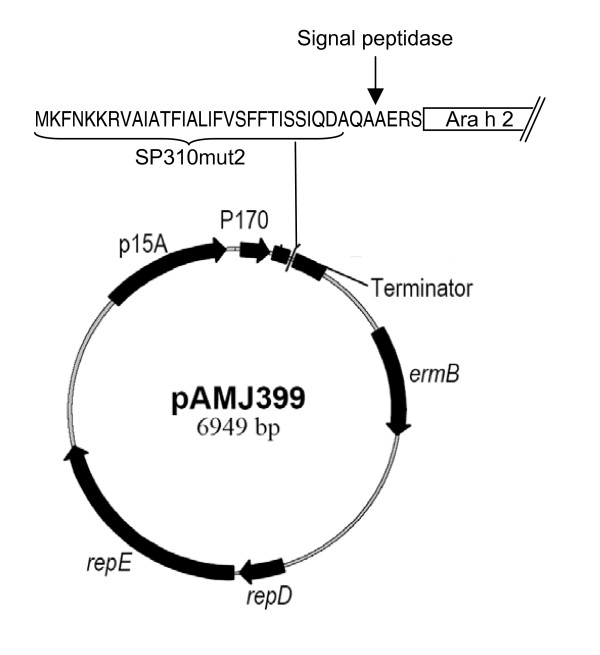
**Anatomy of Ara h 2 expression vector pAMJ399-arah2**. *L. lactis *expression vector pAMJ399. The ara h 2 gene is inserted in fusion with the signal sequence (SP310mut2). The cleavage sire of the signal peptidase and the N-terminal of Ara h 2 is indicated. P170: *L. lactis *P170 promoter, Terminator: transcriptional terminator, *ermB*: gene conferring erythromycin resistance, *repD repE*: *L. lactis *replication unit, p15A: *E. coli *replication region.

**Figure 2:**
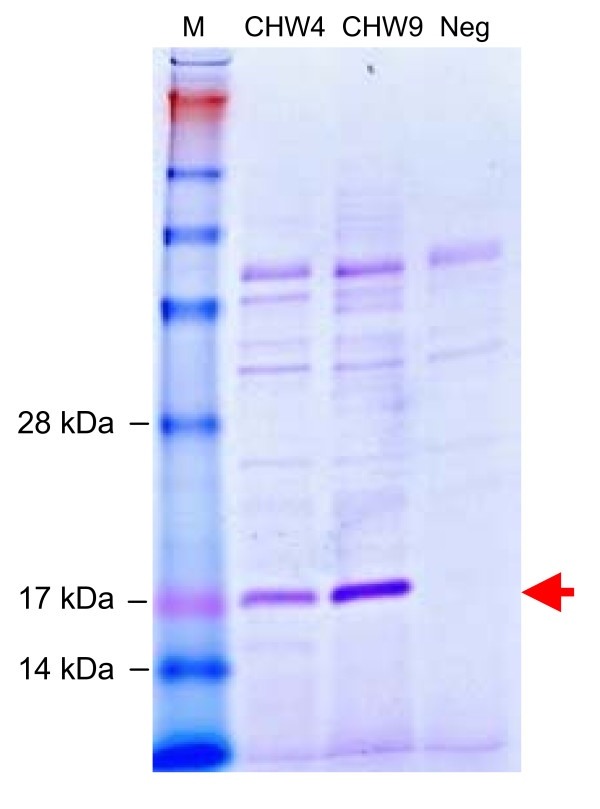
**Expression analysis**. SDS-PAGE of culture supernatants of *L. latis *strain CHW4 and CHW9 harbouring the pAMJ399-arah2 and *L. lactis *strain MG1363 containing plasmid pAMJ399 as negative control (Neg). A clear band of 17 kDa represents secreted rAra h 2.

**Figure 3:**
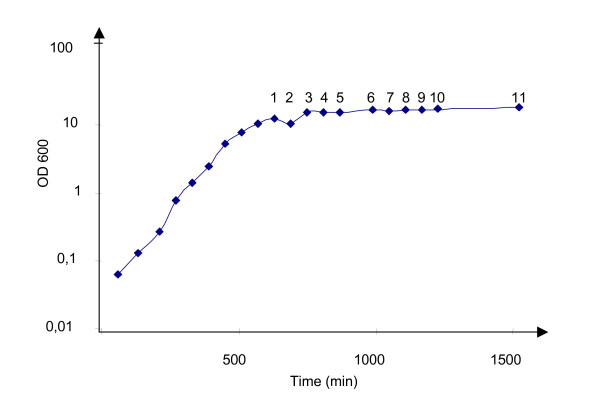
**Growth profile of Ara h 2 producing *L. lactis *CHW9 during fermentation**. Cell densities plotted against time of fermentation. Samples are taken every hour as indicated by numbers.

**Figure 4:**
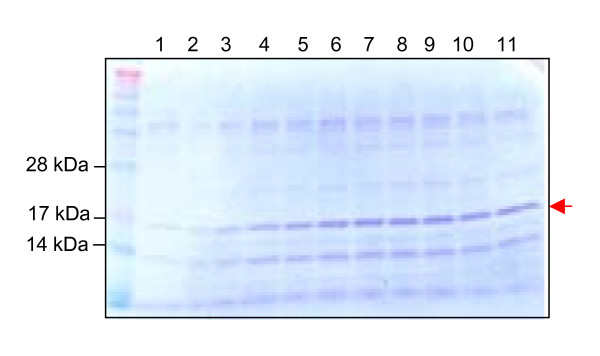
**Culture samples from fermentation**. Crude samples of supernatants corresponding to sample 1–11. Arrow indicates rAra h 2.

To support a high yield expression of the 17 kDa Ara h 2 protein, the gene was chemically synthesised and codon optimized to use most abundant tRNAs of *L. lactis*. Potential translation inhibiting secondary RNA structures was also deleted from the sequence. The 465 bp synthetic gene was inserted into the pAMJ399 expression vector in translational fusion with the signal sequence (Fig. 1). The signal sequence encodes a 32 amino acid signal peptide leading to protein secretion through the sec-dependent pathway [26]. Upon secretion the signal peptide was cleaved off releasing rAra h 2 with a synthetic N-terminal (AERS) extension to the extra cellular milieu (Fig. 1). Expression of Ara h 2 was first tested in flask experiments using different mutant strain backgrounds. The two strains tested were derivatives of *L. lactis *MG1363, made by chemical mutagenesis, and identified as high secretors of recombinant proteins (data not shown). Supernatants from overnight cultures of CHW4 and CHW9, both harbouring pAMJ399-arah2, were analysed and a distinct band of approximate size of 17 kDa was detected in the Ara h 2 transformed strains opposed to the negative control strain harbouring pAMJ399 without the *Ara*h2 gene inserted (Fig. 2). Although the mutant strains repeatedly produced similar cell densities (OD_600 _= 3), strain CHW9 produced more recombinant product than CHW4 and was selected for further testing in 1 litre batch fermentation. Fermentation using CHW9 and synthetic medium gave a maximum cell density of OD_600 _= 12 (Fig. 3). As the cell density increased and reached transition to stationary phase the P170 promoter was induced and a ~17 kDa secreted product was accumulated in the culture supernatant (Fig. 4). SDS-PAGE analysis of culture supernatant (sample 11) and a molecular weight standard of known concentration was made to estimate the concentration of rAra h 2 to 40 mg/L (data not shown). No intracellular accumulation of rAra h 2 was detected (data not shown), which suggests that the secretion of rAra h 2 is not the rate-limiting step in the protein production.

### Purification of rAra h 2

**Figure 5:**
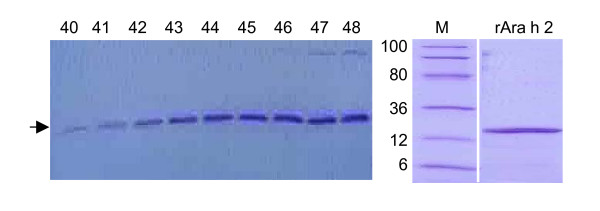
**Purification of rAra h 2 by hydrophic exclusion and size seperation**. Culture supernatant from sample 11 applied to a hydrophic column and eluted by ethanol. 100 fractions are collected and screened. Fractions 40–48 contained the rAra h 2 as indicated by the horizontal arrow. A filtration step excluding proteins above 50 kDa and below 10 kDa resulted in a purified product (depicted as rAra h 2) as analysed by SDS-PAGE in the area of 6–100 kDa (M: Molecular weight).

Although rAra h 2 is secreted to the culture supernatant as the dominant protein in the solution (Fig. 4) a simple purification procedure was established to exclude the native protein secreted by *L. lactis*. Culture supernatant from the fermentation broth was applied to a hydrophobic interaction column and fractions containing the rAra h 2 band were collected (Fig. 5). To separate the rAra h 2 protein from other proteins still present after the hydrophobic exclusion the pooled fractions were purified by two cycles of filtrations to eliminate proteins below 10 kDa and above 50 kDa. The two purification steps resulted in a purified rAra h 2 product as analysed by SDS-PAGE (Fig. 5).

### Characterization of rAra h 2

**Figure 6:**
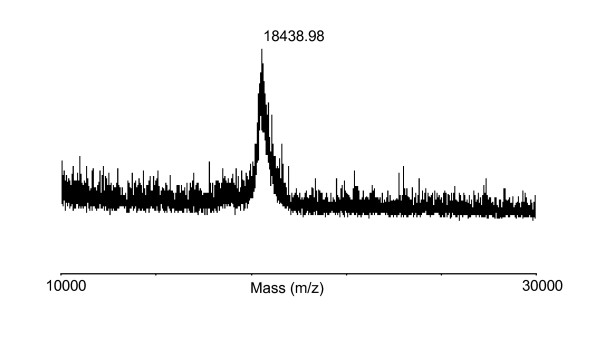
**MS-MALDI TOF analysis of rAra h 2**. Mass spectrum analysis of rAra h 2 showing the dominant peak at 18438,98 Da in the analysed area between 10–30 kDa.

The use of non-native hosts for heterologous protein production can affect the product authenticity due to degradation of the recombinant product, imprecise cleavage of the signal peptide and non-native posttranslational modifications. Therefore, experiments were set-up to determine if the rAra h 2 corresponded to the theoretical predictions. The molecular weight of rAra h 2 was determined by MALDI-TOF mass spectroscopy and showed a peak at *m/z *18438,98 g/mol (Fig. 6), which corresponds to the theoretical value of Ara h 2 being 18437,46 g/mol. This also corresponds to the more imprecise molecular weight estimate derived from SDS-PAGE. No other dominant protein forms were detected in the tested range of 10–30 kDa. To verify that the pre-protein was precisely cleaved by the *L. lactis *signal peptidase, the rAra h 2 was N-terminally sequenced. The first 20 amino acids were identified as AERSRQQWELQGDRRCQSQL, which match the N-terminal of rAra h 2 and the *in silico *prediction of the cleavage site (see additional file 1). In summary, *L. lactis *secreted a full length rAra h 2, which corresponds to the theoretical molecular weight. The rAra h 2 does however contain a four amino acid extension (AERS) originating from the SP310mut2 signal peptide, which is not present in the native Ara h 2.

### Immunochemical reactivity of rAra h 2

**Figure 7:**
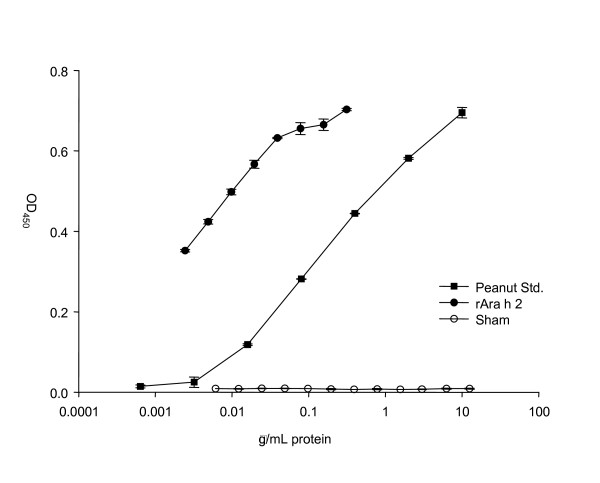
**Immune reactivity of rAra h 2**. Sandwich ELISA using sera from rabbits immunized with purified native Ara h 2. Concentrations on the primary axis refers to protein concentration of peanut extract, rAra h 2, and nuclease. The *L. lactis *produced rAra h 2 was run in two-fold dilutions as was the negative protein control (an *L. lactis *produced nuclease). Curve Std. peanut: extract of Ara h 2 from natural source, curve rAra h 2: *L. lactis *produced rAra h 2, curve Sham: *L. lactis *produced nuclease. Error bars represent SEM of duplicate determinations.

Bacteria lack the post translational machinery responsible for protein modifications like glycosylation and phosphorylation. *L. lactis *may therefore produce an rAra h 2 with predicted amino acid composition, but with a different conformation than that of native Ara h 2. Therefore the immunological equivalence of rAra h 2 and Ara h 2, isolated from a natural source, was compared by ELISA using rabbit antisera raised against purified, native Ara h 2. The rAra h 2 showed strong serum-reactivity compared to the control nuclease protein produced by a similar *L. lactis *strain (Fig. 7). The immune reactivity was therefore related to rAra h 2 and not to other native proteins produced by *L. lactis *and still present in the rAra h 2 solution. The cross-reactivity of rAra h 2 with sera raised by native Ara h 2 was evidenced by perfect parallelism with a peanut extract indicating that most if not all antibody epitopes of native Ara h 2 is present in rAra h 2 (Fig. 7). This demonstrates the immunological activity of the *L. lactis *produced rAra h 2. However, as the immunochemical reactivity of rAra h 2 was measured using rabbit anti-Ara h 2 antisera the presence of human allergenic IgE epitopes in rAra h 2 cannot be determined by this experiment.

## Discussion

Some of the problems associated with allergen immunotherapeutics based on natural sources can be overcome using gene engineering and recombinant production of allergens. The increasing number of sequenced genomes allows for uncomplicated cloning of the allergen encoding cDNA and expression to be done in suitable organisms. Synthetic genes encoding allergens can be synthesised at an affordable prize and be optimized for the specific expression host to support high yield production. Furthermore, in a recombinant production strategy it is relatively simple to engineer hypoallergens with weaker IgE binding epitopes [12,23], which can lower the side effects associated with immunotherapy. Natural allergen products can be heterogeneous from batch to batch and contain undesired and unknown substances. Recombinant production can support a standardised and defined allergen production. However, recombinant production relies on the use of non-native hosts, which may affect the conformational features of the allergen protein. An altered protein conformation or posttranslational modifications like glycosylation or disulfide bond formation can influence the immunereactivity.

In this study we tested *L. lactis *for production of a major peanut allergen. The advantages of *L. lactis *include its food grade status, lack of endotoxins, and high protein secretion capacities. Furthermore, the genetic tools for generation of allergen variants and high trough put screening are well developed.

To ensure high level expression of rAra h 2 the gene was synthetically designed and optimized for expression in *L. lactis*. Furthermore, a high copy number pAMJ399 expression vector was used to increase the gene dosage and maximise rAra h 2 production. Both CHW4 and CHW9 mutant strains efficiently secreted a rAra h 2 of expected molecular mass. The tendency of higher rAra h 2 expression of CHW9 compared to CHW4 cannot be pinpointed to any known mutation as both strains are from a library of chemically and randomly mutagenesised strains and selected as high protein-secretors. The performance of *L. lactis *strain CHW9 with pAMJ399-arah2 was tested in batch fermentation with yield of biomass of OD_600 _= 12. The successful production of 40 mg/L of recombinant Ara h 2 is to our knowledge the highest reported yield in a microbial-based production of Ara h 2. The secretion of rAra h 2 to a supernatant with few native proteins simplifies the downstream processing and purification. Indeed, a two-step purification using hydrophic exclusion and size separation resulted in a pure product as analysed by SDS-PAGE. The potential of adding purification tags could be applied and may ease purification even more. However, a protein tag adds an extra processing step of tag removal by protease cleavage.

Allergens may be complex structures and need higher organisms than prokaryotes for production of a proper folded and modified allergen. In addition, recombinant production can lead to product degradation due to proteolytic activity or premature termination of translation. The protein was therefore further characterized. SDS-PAGE analysis of culture supernatant showed only one band corresponding to the 17 kDa of rAra h 2 and suggests that no truncated forms of the protein were produced (Fig. 2). Furthermore, mass spectrometry analysis of non-digested rAra h 2 gave a molecular mass comparable to the predicted amino acid composition (Fig. 6). This is also in accordance with native Ara h 2, which is without posttranslational modifications like glycosylation and phosphorylation [27]. However, native Ara h 2 contain four conserved disulphide bridges [28], but the *L. lactis *produced rAra h 2 is free of disulphide bridges as observed by comparing the migration length of reduced and non-reduced rAra h 2 samples on SDS-PAGE (data not shown). Lack of intramolecular disulphide formation during protein synthesis in *L. lactis *has been reported [29], which is in accordance with the fact that no putative thiol-disulphide oxidoreductases has been identified in *L. lactis *so far. However, spontaneous formation of disulphide bonds may explain how *L. lactis *can produce biological active proteins where S-S bridges are essential eg. the IL-12 cytokine [30]. The N-terminal of rAra h 2 was also characterized. The native signal peptide of Ara h 2 was interchanged by the *L. lactis *optimized signal peptide SP310mut2. This ensured efficient secretion and correct processing of the signal peptide. Indeed, SP310mut2 was cleaved as predicted (see additional file 1) releasing a rAra h 2 with native amino acid composition, but with a synthetic AERS N-terminal extension.

Although the capacity of *L. lactis *to secrete high levels of rAra h 2 is attractive the biological activity of rAra h 2 is essential. To compare the immunoreactivity of the rAra h 2 to that of natural Ara h 2 an ELISA was established. The *L. lactis *produced rAra h 2 showed similar immune reactivity as that of natural Ara h 2. This indicates that most of the antibody epitopes are retained in the recombinant Ara h 2. However, to establish biological equivalence with native Ara h 2 a large panel of sera from clinically allergic patients should be tested by radioallergosorbent test (RAST) and in basophil histamine release experiments. Such clinical documentation was, however, beyond the scope of the present study.

The use of rAra h 2 with wild type amino acid composition for immunotherapy may be associated with side effects due to its high immunereactivity. By site directed mutagenesis Ara h 2-hypoallergens with weaker IgE epitopes may be obtained. Such hypoallergens with few amino acid changes will have close resemblance to rAra h 2 and should be as highly expressed as the wild type allergen in *L. lactis*. However, the lack of disulphide bonds in rAra h 2 may be sufficient to lower its IgE binding capacity as deletion of S-S bridges has been shown to disrupt IgE eitopes and lower the immunereactivity of proteins [13]. If this is the case for the *L. lactis *produced rAra h 2 is to be shown. The rAra h 2 may also be a valuable tool for component resolved allergy diagnosis.

*L. lactis *has also been used for the *in vivo *delivery of therapeutic components. Steidler *et al*. showed that *L. lactis *could deliver active IL-10 to the gastrointestinal tract by orally feeding mice with recombinant bacteria [31], which recently has been tested in a clinical study with positive results on patients suffering from Crohn's disease [32]. *In situ *delivery of allergy components has also been evaluated using live recombinant lactic acid bacteria. Intranasal administration of birch pollen Bet v 1-secreting-*L. lactis *reduced allergen specific IgE in a mouse model [33]. Recently, immunomodulatory *L. casei *has been developed to secrete the important milk allergen β-lactoblobulin [34], which currently is being tested as a therapeutic treatment in an allergy mouse model. The rAra h 2 producing strain presented in this study could also be used for *in vivo *allergen delivery, where the intrinsic adjuvant capacity of lactic acid bacteria is exploited.

## Conclusion

Recombinant allergen production can alleviate some of the problems associated to allergen products based on natural sources. Recombinant allergens represent an important tool in component resolved allergy analysis, development of engineered hypoallergens, and production of allergens with defined composition and purity. Peanut allergy is an example where recombinant allergens can provide material for component resolved analysis of the allergic response and guide the engineering of safer hypoallergens. We showed that *L. lactis *can sustain high-level expression of 40 mg/L of full-length rAra h 2 and developed a simple purification protocol. The produced rAra h 2 was biologically active and showed immune cross reactivity with natural Ara h 2. The relatively simple production and purification process of rAra h 2 makes *L. lactis *an interesting expression system for the production of the major allergens present in peanuts.

## Methods

### Bacteria and media

*L. lactis *was grown at 30°C in M17 medium (Oxoid, Hampshire, United Kingdom) supplemented with 0,5% glucose (GM17). When appropriate, 1 μg/mL erythromycin (Merck, Darmstadt, Germany) was added. Fed batch fermentation was done using 1 L *L. lactis *culture, which was cultivated for 25 h in LM3-30 [35] in 2 L fermentors at 30°C. 5 M KOH was automatically added to maintain pH at 6 and the agitation rate was set at 300 rpm. Growth was monitored by measuring OD_600._

### Construction of rAra h 2 producing *L. lactis*

Plasmid DNA from *L. lactis *was prepared as described [36]. *L. lactis *was made electro-competent and transformed as described [37]. DNA restriction and modification enzymes, New England Biolabs (Beverly, MA) were used as recommended. The gene encoding Ara h 2 was synthetically manufactured by Geneart (Regensburg, Germany) using the Geneoptimizer program for optimal expression in *L. lactis*. The amino acid composition of Ara h 2 was from NCBI locus number AAM78596 where the native 18 aa signal peptide of Ara h 2 (LTILVAPALFLLAAHASA) was replaced by the SP310mut2 *L. lactis *signal peptide. The 459 bp gene was added terminally *Bgl *II and *Sal *I restriction enzyme recognition sites by the manufacturer for cloning in pAMJ399 in fusion to the signal sequence SP310mut2 (Fig. 1). *L. lactis *clones with pAMJ399 harbouring the Ara h 2 gene in *Bgl *II and *Sal *I sites were verified using PCR and primers specific for the synthetic Ara h 2 gene. A PCR positive clone was tested for expression of rAra h 2 by overnight growth in GM17 supplemented with 1 μg/mL erythromycin. This plasmid was named pAMJ399-arah2. Transformation of the mutant strains PSM565 and DOL7 with pAMJ399-arah2 resulted in CHW4 and CHW9, respectively. Supernatants from overnight grown cultures were analysed by SDS-PAGE and band densities of rAra h 2 were compared.

### Expression and purification of rAra h 2

Samples were taken every hour from fermentation and divided into cell pellet and supernatant by centrifugation at 8000 × g for 5 min and frozen at -20°C. Culture supernatants were TCA precipitated and analysed. Cell pellet was lysed using glass beads and cellular debris was removed by centrifugation at 15000 × g for 15 min at 4°C and the intracellular protein fraction was isolated as the soluble fraction. Intra- and extracellular protein fractions were analysed using 14% SDS-PAGE. Culture supernatant from the end of fermentation was adjusted with (NH_4_)_2_SO_4 _to 1 M and applied to a phenyl sepharose column (GE-Heathcare, Hillerød, Denmark) and washed in buffer A (Tris-Cl 50 mM, (NH_4_)_2_SO_4 _1 M, pH 8). Protein was eluted with buffer B (Tris-Cl 50 mM, pH 8) and collected automatically in 5-mL fractions using Frac-100 (Pharmacia Biotech, Freiburg, Germany). Fractions were analysed by SDS-PAGE. Selected fractions containing rAra h 2 were pooled and desalted by a PD-10 Sephadex column (Sigma-Aldrich, Brøndby, Denmark) and small and large protein impurities were removed by Amicon ultra-4 columns (Millipore, Billerica, MA) with cut off values of 10 kDa and 50 kDa.

### MALDI-TOF analysis

Molecular weight of rAra h 2 was determined by matrix-assisted laser desorption ionization time-of flight mass spectrometry (MALDI-TOF MS). 0,5 μL of purified protein (0,5-1 ng) was applied on a tip and mixed with 1 μL of 1,3 mg/mL of HABA (2-(4-Hydroxyphenylazo)benzoic acid from Sigma-Aldrich) dissolved in H_2_O/CH_3_CN (1:1) solution. The mass spectra were obtained on a Voyager-DE™ Pro instrument (Applied Biosystems, Weiterstadt, Germany) operating at an accelerating voltage of 20 kV in the linear mode with the delayed extraction setting. Recorded data were processed using Data Explorer™ 4.0 software.

### N-terminal sequencing

Purified rAra h2 was loaded onto a 14% SDS-PAGE gel. After separation the protein was electro blotted onto a PVDF membrane at 175 mA for 1 h in a Semi Dry Blotter II (Kem-En-Tec, Tåstrup, Denmark) using a 10 mM CAPS blotting solution containing 6% methanol. The membrane was stained with 0,1% Coomassie Brilliant blue in 1% acetic acid and 60% methanol for 1 min and destained for 5 min in 40% methanol until the background was light purple/blue. The membrane was rinsed in deionised water and air-dried. The relevant protein band was excised and subjected to N-terminal sequencing using Edman degradation on a Procise Protein Sequencer494 (Applied Biosystems).

### ELISA

A 96-well ELISA plate was coated overnight at 4°C with rabbit antiserum raised against purified and native Ara h 2 (a kind gift from Dr. W-M Becker, Forschungszentrum Borstel, Germany). Excess coating buffer was discarded and blocking buffer (1 mg/mL gelatine in washing buffer) was added to the wells and the plate was placed on a shaker at room temperature for 1 h. The plate were washed 3 times in washing buffer (NaCl 80,0 g/L, KCl 2,0 g/L, KH_2_PO_4 _2,0 g/L, Na_2_HPO_4 _14,4 g/L, Tween 206 g/L, pH 7,4). A 100 μL of positive control and 100 μL of rAra h2 were added to the wells and serial diluted and incubated 2 h at 37°C. Peanut extract served as positive control and was obtained using unshelled peanuts (Brüder Kunz GmbH), which were grounded in a mortar with a pestle and prepared as described [38] and kept at -20°C. Negative control in ELISA was TE buffer and culture supernatant of a *L. lactis *MG1363 strain expressing the *Staphyloccous aureus *nuclease (21 kDa). After incubation the plate were washed three times in washing buffer. Subsequently, 100 μL of biotinylated Ara h 2 specific antibody, diluted 1:2500, was added to each well and plates were incubated for 2 h at 37°C. Extravidin diluted 1:5000 in washing buffer was applied to each well (100 μL) and left at room temperature on a shaker for 30 min. Following 3 washes in washing buffer the substrate (o-Phenylenediamine dihydrochloride tablets dissolved in water added hydrogen peroxide) was added and the plate incubated 10–15 min. at room temperature in the dark. The reaction was stopped with 150 μL stop solution. The absorbance at 450 nm was measured.

## Competing interests

The author(s) declare that they have no competing interests.

## Authors' contributions

JG designed and supervised the experiments. JG drafted the manuscript. HW made most of the experiments and edited the manuscript. SM and CW gave helpful contributions on expression and fermentation experiments. HF gave valuable know-how on allergens. KK supervised the mass spectrometry analysis. LP supervised the immunological studies of rAra h 2 and edited the manuscript.

## Supplementary Material

Additional file 1 **Signal peptide prediction**. The amino acid sequence of rAra h 2 is submitted to the prediction server  using the gram positive bacteria as settings. The C-score is the cleavage site score. For each position in the submitted sequence, a C-score is reported, which should only be significantly high at the cleavage site. Y-max is a derivative of the C-score combined with the S-score resulting in a better cleavage site prediction than the raw C-score alone. This is due to the fact that multiple high-peaking C-scores can be found in one sequence, where only one is the true cleavage site. The cleavage site is assigned from the Y-score where the slope of the S-score is steep and a significant C-score is found. The *S-mean *is the average of the S-score, ranging from the N-terminal amino acid to the amino acid assigned with the highest Y-max score, thus the S-mean score is calculated for the length of the predicted signal peptide. Click here for file

## References

[B1] Norman PS (2004). Immunotherapy: 1999–2004. J Allergy Clin Immunol.

[B2] Wilson DR, Torres LI, Durham SR (2003). Sublingual immunotherapy for allergic rhinitis. Cochrane Database Syst Rev.

[B3] Dahl R, Kapp A, Colombo G, de Monchy JG, Rak S, Emminger W (2006). Efficacy and safety of sublingual immunotherapy with grass allergen tablets for seasonal allergic rhinoconjunctivitis. J Allergy Clin Immunol.

[B4] Khinchi MS, Poulsen LK, Carat F, Andre C, Hansen AB, Malling HJ (2004). Clinical efficacy of sublingual and subcutaneous birch pollen allergen-specific immunotherapy: a randomized, placebo-controlled, double-blind, double-dummy study. Allergy.

[B5] Spangfort MD, Larsen JN (2006). Standardization of allergen-specific immunotherapy vaccines. Immunol Allergy Clin North Am.

[B6] Valenta R, Niederberger V (2007). Recombinant allergens for immunotherapy. J Allergy Clin Immunol.

[B7] Hoffmann-Sommergruber K, Susani M, Ferreira F, Jertschin P, Ahorn H, Steiner R (1997). High-level expression and purification of the major birch pollen allergen, Bet v 1. Protein Expr Purif.

[B8] Ledesma A, Moral V, Villalba M, Salinas J, Rodriguez R (2006). Ca2+-binding allergens from olive pollen exhibit biochemical and immunological activity when expressed in stable transgenic Arabidopsis. FEBS J.

[B9] Chapman MD, Smith AM, Vailes LD, Arruda LK, Dhanaraj V, Pomes A (2000). Recombinant allergens for diagnosis and therapy of allergic disease. J Allergy Clin Immunol.

[B10] Poltl G, Ahrazem O, Paschinger K, Ibanez MD, Salcedo G, Wilson IB (2007). Molecular and immunological characterization of the glycosylated orange allergen Cit s 1. Glycobiology.

[B11] Olsson S, van Hage-Hamsten M, Whitley P, Johansson E, Hoffman DR, Gafvelin G (1998). Expression of two isoforms of Lep d 2, the major allergen of Lepidoglyphus destructor, in both prokaryotic and eukaryotic systems. Clin Exp Allergy.

[B12] Vrtala S, Focke-Tejkl M, Swoboda I, Kraft D, Valenta R (2004). Strategies for converting allergens into hypoallergenic vaccine candidates. Methods.

[B13] Smith AM, Chapman MD (1996). Reduction in IgE binding to allergen variants generated by site-directed mutagenesis: contribution of disulfide bonds to the antigenic structure of the major house dust mite allergen Der p 2. Mol Immunol.

[B14] van Hage-Hamsten M, Kronqvist M, Zetterstrom O, Johansson E, Niederberger V, Vrtala S (1999). Skin test evaluation of genetically engineered hypoallergenic derivatives of the major birch pollen allergen, Bet v 1: results obtained with a mix of two recombinant Bet v 1 fragments and recombinant Bet v 1 trimer in a Swedish population before the birch pollen season. J Allergy Clin Immunol.

[B15] Emmett SE, Angus FJ, Fry JS, Lee PN (1999). Perceived prevalence of peanut allergy in Great Britain and its association with other atopic conditions and with peanut allergy in other household members. Allergy.

[B16] Sicherer SH, Munoz-Furlong A, Burks AW, Sampson HA (1999). Prevalence of peanut and tree nut allergy in the US determined by a random digit dial telephone survey. J Allergy Clin Immunol.

[B17] Woods RK, Thien F, Raven J, Walters EH, Abramson M (2002). Prevalence of food allergies in young adults and their relationship to asthma, nasal allergies, and eczema. Ann Allergy Asthma Immunol.

[B18] de Leon MP, Rolland JM, O'Hehir RE (2007). The peanut allergy epidemic: allergen molecular characterisation and prospects for specific therapy. Expert Rev Mol Med.

[B19] Koppelman SJ, Wensing M, Ertmann M, Knulst AC, Knol EF (2004). Relevance of Ara h1, Ara h2 and Ara h3 in peanut-allergic patients, as determined by immunoglobulin E Western blotting, basophil-histamine release and intracutaneous testing: Ara h2 is the most important peanut allergen. Clin Exp Allergy.

[B20] Stanley JS, King N, Burks AW, Huang SK, Sampson H, Cockrell G (1997). Identification and mutational analysis of the immunodominant IgE binding epitopes of the major peanut allergen Ara h 2. Arch Biochem Biophys.

[B21] Roy K, Mao HQ, Huang SK, Leong KW (1999). Oral gene delivery with chitosan--DNA nanoparticles generates immunologic protection in a murine model of peanut allergy. Nat Med.

[B22] Glenting J, Wessels S (2005). Ensuring safety of DNA vaccines. Microb Cell Fact.

[B23] King N, Helm R, Stanley JS, Vieths S, Luttkopf D, Hatahet L (2005). Allergenic characteristics of a modified peanut allergen. Mol Nutr Food Res.

[B24] Lehmann K, Hoffmann S, Neudecker P, Suhr M, Becker WM, Rosch P (2003). High-yield expression in Escherichia coli, purification, and characterization of properly folded major peanut allergen Ara h 2. Protein Expr Purif.

[B25] Madsen SM, Arnau J, Vrang A, Givskov M, Israelsen H (1999). Molecular characterization of the pH-inducible and growth phase-dependent promoter P170 of *Lactococcus lactis*. Mol Microbiol.

[B26] Ravn P, Arnau J, Madsen SM, Vrang A, Israelsen H (2003). Optimization of signal peptide SP310 for heterologous protein production in *Lactococcus lactis*. Microbiology.

[B27] Lehmann K, Schweimer K, Reese G, Randow S, Suhr M, Becker WM (2006). Structure and stability of 2S albumin-type peanut allergens: implications for the severity of peanut allergic reactions. Biochem J.

[B28] Barre A, Borges JP, Culerrier R, Rouge P (2005). Homology modelling of the major peanut allergen Ara h 2 and surface mapping of IgE-binding epitopes. Immunol Lett.

[B29] van de GM, van der Wal FJ, Kok J, Venema G (1992). Lysozymeexpression in *Lactococcus lactis*. Appl Microbiol Biotechnol.

[B30] Bermudez-Humaran LG, Langella P, Cortes-Perez NG, Gruss A, Tamez-Guerra RS, Oliveira SC (2003). Intranasal immunization with recombinant *Lactococcus lactis *secreting murine interleukin-12 enhances antigen-specific Th1 cytokine production. Infect Immun.

[B31] Steidler L, Hans W, Schotte L, Neirynck S, Obermeier F, Falk W (2000). Treatment of murine colitis by *Lactococcus lactis *secreting interleukin-10. Science.

[B32] Braat H, Rottiers P, Hommes DW, Huyghebaert N, Remaut E, Remon JP (2006). A phase I trial with transgenic bacteria expressing interleukin-10 in Crohn's disease. Clin Gastroenterol Hepatol.

[B33] Repa A, Grangette C, Daniel C, Hochreiter R, Hoffmann-Sommergruber K, Thalhamer J (2003). Mucosal co-application of lactic acid bacteria and allergen induces counter-regulatory immune responses in a murine model of birch pollen allergy. Vaccine.

[B34] Hazebrouck S, Pothelune L, Azevedo V, Corthier G, Wal JM, Langella P (2007). Efficient production and secretion of bovine beta-lactoglobulin by *Lactobacillus casei*. Microb Cell Fact.

[B35] Glenting J, Madsen SM, Vrang A, Fomsgaard A, Israelsen H (2002). A plasmid selection system in *Lactococcus lactis *and its usefor gene expression in *L. lactis *and human kidney fibroblasts. Appl Environ Microbiol.

[B36] O'Sullivan DJ, Klaenhammer TR (1993). Rapid Mini-Prep Isolation of High-Quality Plasmid DNA from *Lactococcus *and *Lactobacillus *spp. Appl Environ Microbiol.

[B37] Holo H, Nes IF (1995). Transformation of *Lactococcus *by electroporation. Methods Mol Biol.

[B38] Poulsen LK, Pedersen MH, Platzer M, Madsen N, Sten E, Bindslev-Jensen C (2003). Immunochemical and biological quantification of peanut extract. Arb Paul Ehrlich Inst Bundesamt Sera Impfstoffe Frankf A M.

