# Supplementary figures and images for: Production of Recombinant Peanut Allergen Ara h 2 using Lactococcus lactis
Source: Microb Cell Fact. 2007 Aug 21;6:28. doi: 10.1186/1475-2859-6-28 (PMC2000909; doi:10.1186/1475-2859-6-28)

SignalP-NN prediction (gram+ networks): Sequence

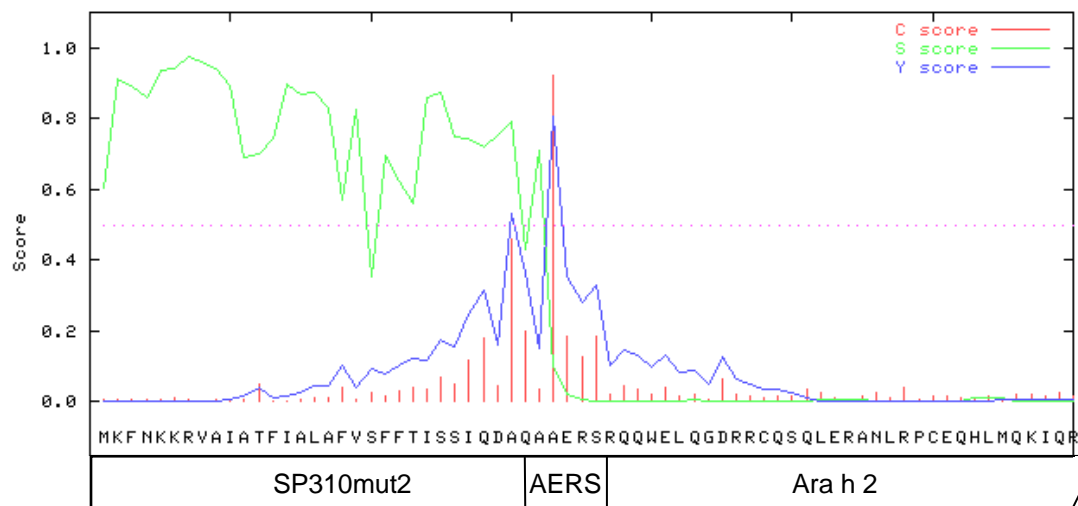

Supplement: Additional file 1 — Signal peptide prediction. The amino acid sequence of rAra h 2 is submitted to the prediction server using the gram positive bacteria as settings. The C-score is the cleavage site score. For each position in the submitted sequence, a C-score is reported, which should only be significantly high at the cleavage site. Y-max is a derivative of the C-score combined with the S-score resulting in a better cleavage site prediction than the raw C-score alone. This is due to the fact that multiple high-peaking C-scores can be found in one sequence, where only one is the true cleavage site. The cleavage site is assigned from the Y-score where the slope of the S-score is steep and a significant C-score is found. The S-mean is the average of the S-score, ranging from the N-terminal amino acid to the amino acid assigned with the highest Y-max score, thus the S-mean score is calculated for the length of the predicted signal peptide. [file 1475-2859-6-28-S1.pdf]
